# A Wave‐Driven Piezoelectrical Film for Interfacial Steam Generation: Beyond the Limitation of Hydrogel

**DOI:** 10.1002/advs.202204187

**Published:** 2022-10-10

**Authors:** Sen Meng, Chun‐Yan Tang, Jie Yang, Ming‐Bo Yang, Wei Yang

**Affiliations:** ^1^ College of Polymer Science and Engineering Sichuan University State Key Laboratory of Polymer Materials Engineering Chengdu Sichuan 610065 P. R. China

**Keywords:** hydrogels, piezoelectricity, water activation, wave‐driven

## Abstract

Solar interfacial vapor generation based on low evaporation energy requirements is an effective technology to speed up water purification under natural sunlight, offering great potential to alleviate the current global water crisis. The external electric field and hydrogel are two independent methods enabling low‐energy water evaporation. However, the complicated external equipment for generating an electric field and the restricted activation area of hydrogels significantly limit their practical application in steam generation. Thus, a piezoelectric fiber membrane is embedded into a highly hydratable light‐absorbing poly(vinyl alcohol) (PVA) hydrogel for synergistic water activation. The integrated evaporator is capable of continuously converting the wave energy reserved in the ocean into electrical energy, activating the water in the hydrogel. It is found that the activation effect leads to an improvement of over 23% compared to a non‐piezoelectric hydrogel evaporator. This work provides an evaporation prototype based on the synergistic water activation of wave‐triggered electricity and highly hydratable hydrogel.

## Graphical abstract

A self‐powered solar water purification technique containing an all‐in‐one composite film evaporator composed of light‐absorbing hydrogel and piezoelectric fiber membrane, which triggers water activation in the hydrogel to synergistically combat the intrinsically high energy demand for water vaporization, thus delivering a high steam generation rate of 2.702 kg m^−2^ h^−1^ with an improvement of ≈23% compared to the non‐piezoelectric evaporator under one sun, is presented.

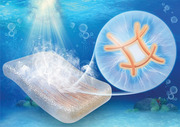

## 1 Introduction

Large water demands driven by global water scarcity and growing population open up new opportunities for developing efficient clean water extraction materials and systems.^[^ ^1^, ^2^, ^3^, ^4^, ^5^, ^6^, ^7^, ^8^, ^9^ ^]^ Solar‐driven interfacial water purification, an emerging and enticing technique, localizes solar thermal energy at air‐liquid interface to continuously produce clean water through the evaporation of seawater.^[^ ^10^, ^11^, ^12^, ^13^, ^14^, ^15^, ^16^, ^17^, ^18^ ^]^ Water evaporation, i.e., the phase transition of water from liquid to gas, is a highly energy‐intensive process due to that each water molecule in the liquid phase is confined in a constantly changing network of hydrogen bonds with high energy density.^[^ ^19^, ^20^, ^21^ ^]^ Over the past few years, enthusiastic efforts have been devoted to lowering the energy demand for water evaporation.^[^ ^22^, ^23^, ^24^, ^25^ ^]^ For example, hydrogel materials with abundant hydrophilic functional groups can bond with water molecules through non‐covalent hydrogen bonding interactions and intervene the intermolecular force and hydrogen bonding network, reducing the water vaporization enthalpy and activating the water evaporation.^[^ ^26^, ^27^, ^28^ ^]^ Some previous works targeting the intensive hydration capacity of hydrogels seem to become an insurmountable bottleneck in the evaporation rate. Nevertheless, its activation effect is restricted with confined molecular chain regions of hydrophilic polymer inside the hydrogel,^[^ ^29^, ^30^ ^]^ which trammels the re‐breakthrough of its water activation capacity, generating a limited evaporation rate. In addition, an electrostatic field or alternating electric field can also modify the network of water molecules.^[^ ^31^, ^32^ ^]^ The polarization and Lorentz force induced by the electric field can accelerate water molecular movement and enhance the intermolecular collision possibilities,^[^ ^33^, ^34^ ^]^ weakening the hydrogen bonding strength and reducing the energy barrier of phase change of water. However, directly applying the external electric field to activate water will inevitably increase enormous energy input and economic cost, which are against the original intention of green and sustainable development.

Notably, abundant renewable and green energy resources exist in the ocean, including tidal energy, ocean current energy, wave energy, and thermal energy.^[^ ^1^, ^35^, ^36^ ^]^ Among these resources, wave energy is the most widely distributed energy source with the highest energy flux density, coincidentally locating at the water‐air interface.^[^ ^37^, ^38^ ^]^ In this regard, piezoelectric materials attract our attention for electrical energy supply, which can instantly convert mechanical stimulus into electrical energy and release it.^[^ ^39^, ^40^, ^41^ ^]^ The reciprocating forces and potential energy differences contained in ocean waves can be converted into electricity by piezoelectric materials. The water around the piezoelectric materials is activated by this electric field, giving rise to a low vaporization enthalpy. Inspired by this, the integration of hydrogel and piezoelectric materials can be an ideal candidate for an efficient water evaporator in terms of regional attenuated vaporization enthalpy and sustained low energy consumption.

**Figure 1:**
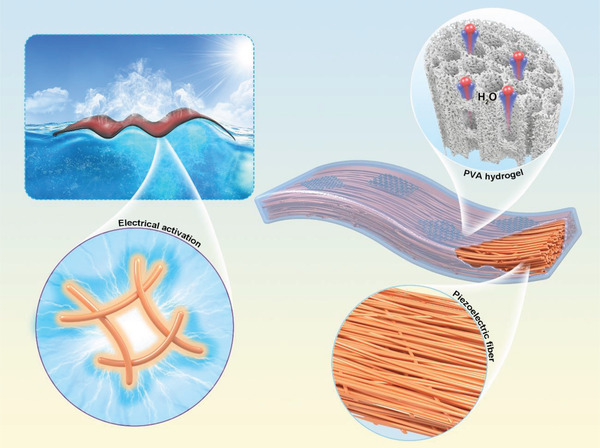
Schematic illustration of the solar interfacial vapor generation system based on a piezoelectric composite film evaporator consisting of an anisotropic fiber membrane and hydratable light‐absorbing hydrogel for water purification, in which the highly hydratable light‐absorbing PVA hydrogel is customized with an oriented pore structure to enhance water transport. Under solar irradiation, this composite film evaporator can continuously convert wave energy reserved in the ocean into uninterrupted electrical energy, reactivating the water in the hydrogel that has been activated by highly hydrophilic polymer networks. The rGO in the hydrogel efficiently harvests solar energy and converts it into thermal energy to enable the water that has been dually activated to evaporate, giving rise to a high evaporation rate.

In this work, an all‐in‐one composite film evaporator composed of light‐absorbing hydrogel and piezoelectric fiber membrane was designed to synergistically combat the intrinsically high energy demand for water vaporization, as illustrated in **Figure** **1**. The hierarchically structured light‐absorbing hydrogel with highly hydratable polymer networks and oriented water transport channels was prepared by interspersing reduced.

graphene oxide (rGO) light absorbers into a matrix consisting of poly(vinyl alcohol) (PVA) and tannic acid (TA) through an ice‐templating‐assisted in situ gelation strategy. The piezoelectric fibers prepared by electrostatic spinning of poly(vinyl fluoride‐trifluoroethylene) (PVDF‐TrFE) exhibit a highly aligned and anisotropic structure, which can be maximally stimulated by bending in the orientation direction of the fibers to generate electrical energy. The monolithic composite film evaporator is localized on the water surface along the direction of wave reciprocating motion. The perceived mechanical stimulus, such as direct water impact force and bending deformation with the wave, are instantly converted into electrical energy, reactivating the water in the hydrogel that has been activated by highly hydrophilic polymer networks. Consequently, compared to a non‐piezoelectric evaporator, the resulting film evaporator composed of the hydratable hydrogel with piezoelectric materials deliver a lower equivalent evaporation enthalpy of 1.077 kJ g^−1^ and a high steam generation rate of 2.702 kg m^−2^ h^−1^ with an improvement of ≈23% under one sun. Such a synergistic water activation strategy of hydrogel and electrical energy generated from the ocean waves provides a more realistic solution for effective and low‐energy solar water purification of practical significance.

## 2 Results and Discussion

### 2.1 Preparation and Morphology of the Composite Film Evaporator

**Figure 2:**
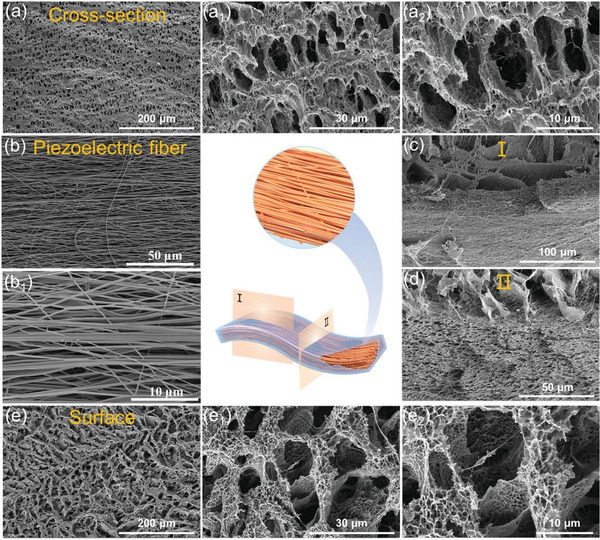
Morphology of the composite film evaporator. a) Cross‐sectional SEM images of the hydrogel component in freeze‐dried composite film evaporator with different magnifications. b) Surface SEM images of PVDF‐TrFE piezoelectric fiber membranes with different magnifications. c) Longitudinal and d) cross‐sectional SEM images at the interface of the hydrogel and the piezoelectric fiber in the freeze‐dried composite film evaporator. e) Surface SEM images of the hydrogel component in freeze‐dried composite film evaporator with different magnifications.

The polymer networks comprising PVA and TA in the composite film evaporator are constructed by in situ co‐gelation method. Worthy of note is that an anisotropic pore structure is further customized using a directional freeze‐casting strategy during the gelation process. Additionally, the piezoelectric fiber membrane in the composite evaporator is encapsulated inside the hydrogel prior to its gelation, as shown in Figure S1, Supporting Information. Scanning electron microscopy (SEM) images show the cross‐sectional morphology of the hydrogel component in the freeze‐dried composite evaporator (**Figure** **2**a). Pores with a diameter of ≈6.6 µm are uniformly distributed in the composite evaporator and present a typical anisotropic feature induced by ice crystals. The water channels oriented along the out‐of‐plane direction of the evaporator are established to accelerate water transportation. In addition, pores at the nanoscale (hundreds of nanometers) are equally uniformly distributed in the composite evaporator (Figure 2a, _1_,a_2_), manifesting a typically hierarchical structure. Figure 2b shows the morphology of piezoelectric PVDF‐TrFE fibers with a highly anisotropic structure, which were arranged via electrostatic spinning with a high collecting speed of 3000 rpm. The piezoelectric fiber membrane and the external hydrogel are a physical combination, and no change in the physical structure and chemical composition of the external hydrogel is observed after introducing the piezoelectric fiber membrane The interface between the hydrogel and the fiber in the composite evaporator is shown in Figure 2c,d, and no significant separation can be observed. The hydrogel adheres closely to the piezoelectric fibers, beneficial for facilitating the response of the fibers to external stimulus and reactivating the water in the hydrogel. Figure 2e shows the surface morphology of the composite evaporator with a hierarchical porous structure analogous to the interior of the hydrogel, confirming the effectiveness of the co‐gelation method to fabricate hydrogels with a stable and homogeneous structure.

### 2.2 Structure and Performance Optimizations of the Hydrogel in the Composite Film Evaporator

The pore structure of the hydrogels in the composite film evaporator is derived from the network of PVA and TA, and TA component can increase the mechanical property and stability of the PVA network through intensive hydrogen bonds (see details in section S1.1, Supporting Information).^[^ ^42^ ^]^ A co‐solvent system of water and glycerol was chosen to increase the solubility of TA in water. In addition, glycerol can improve the crystallization ability of PVA, forming a stable hierarchical network structure after only one freeze‐thaw process. To analyze the effect of glycerol on the network structure, the individual hydrogel samples with various water/glycerol weight ratios from 1:1 and 1:0.429 to 1:0.111 are prepared and abbreviated as 5–5, 7–3, and 9–1, respectively. Moreover, the 9–1 sample with an additional ice‐templating control process is noted as 9–1–C.

**Figure 3:**
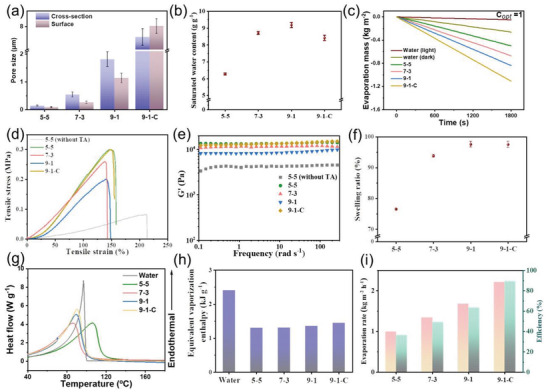
a) Average pore size in the hydrogels with different water/glycerol ratios. b) The saturated water content of the hydrogels with different water/glycerol ratios. c) The evaporation mass of pure water for different hydrogels under one sun in comparison to water evaporation without any hydrogels as a blank control. d) Tensile stress‐strain curves of the hydrogels with different water/glycerol ratios. e) Oscillatory frequency sweeps of the hydrogels with different water/glycerol ratios with 0.1% strain at 25 °C. f) Swelling ratio of the hydrogels with different water/glycerol ratios during the water absorption process. g) Typical DSC melting curves of pure water and hydrogels with different water/glycerol ratios. h) Equivalent vaporization enthalpies of pure water and the hydrogels with different water/glycerol ratios. i) Water evaporation rates and solar steam efficiencies of the hydrogels with different water/glycerol ratios.

Figure S2a‐c shows the cross‐sectional and surface morphology of the 5–5, 7—3, and 9–1 hydrogels, respectively, and the relevant pore sizes are presented in **Figure** **3**a. Along with the rise of the water composition in the co‐solvent, the entanglement of PVA molecular chains becomes weakened, illuminating a tendency to increase the pore size. It is worth noting that the pore structure of the hydrogel on the surface cannot maintain consistency with the interior, which could be attributed to the hydrophobic surface properties of the mold do not match the properties of the PVA (see details in Section S1.2, Supporting Information). As a result, a super‐hydrophilic coating was used to instantly reverse the surface properties of the mold. After the modification, the 9–1 sample with the largest pore size (≈1.82 µm) shows an isotropic and interlinked three‐dimensional network structure (Figure S3, Supporting Information). The saturated water content of hydrogels is shown in Figure 3b. After increasing the proportion of water in the co‐solvent, the significantly raised saturated water content facilitates the water transport and the water supply during evaporation. In addition, the vapor generation performance of the three hydrogels is determined by the mass change of pure water under one sun (Figure 3c). Unfortunately, the surfaces of all three hydrogels presented an integrally dry state owing to that water cannot be instantly replenished to the evaporating surface through the pores in the hydrogels. A 9–1–C hydrogel with a larger pore size and anisotropic pore structure was prepared using a directional freeze‐casting strategy to significantly advance the water transportation in the hydrogel, leading to a prominent increase in evaporation rate (Figure 3c). In addition, a detailed visual comparison of the water transport performance for different hydrogels is shown in Section S1.3, Supporting Information, clearly demonstrating the excellent water transport capability of the 9—1–C hydrogel.^[^ ^43^ ^]^

In addition, ice‐templating‐assisted gelation strategy increases the crystallinity of PVA hydrogels due to its additional treatment at extremely low temperatures of −100 °C during directional freezing,^[^ ^44^, ^45^, ^46^ ^]^ which has been corroborated by Differential scanning calorimetry (DSC) results (see details in Section S1.4, Supporting Information). Specifically, with the increase of the water content in the co‐solvent system, the intermolecular interaction of PVA is weakened, simultaneously reducing the crystallinity and deteriorating PVA network stability. Notably, the ice‐templating‐based 9—1–C hydrogel manifests a reascension in crystallinity compared to the 9–1 hydrogel, and thus forming a more well‐organized and well‐defined PVA nanoscale network as shown in SEM images (Figure 2e_1_,e_2_) and resulting in a slight decrease in the saturated water content (Figure 3b). To analyze the mechanical properties, the tensile strain−stress curves and the dynamic mechanical analyses of different PVA hydrogels were performed, as shown in Figure 3d,e. The introduction of TA significantly increased the tensile strength and the storage modulus (G′) of the hydrogels. Similar to the crystallization behavior of PVA hydrogels, a reascension in the mechanical property for the 9—1–C hydrogel is observed, which is beneficial to the stability of the hydrogel network structure under the natural ocean waves.

The glycerol composition in the composite film can be completely removed during the water absorption process after the hydrogel gelation, and the swelling ratio defined as the volume change of the hydrogel during water absorption is shown in Figure 3f. The 9—1–C hydrogel with almost 100% change ratio exhibits excellent morphological structure retention. After integrating with the piezoelectric fiber membrane, the highly aligned structure of the internal fiber is maintained during the water absorption process of the 9—1–C hydrogel. To evaluate the evaporation efficiency of different hydrogels, the equivalent water vaporization enthalpy was determined by DSC measurement (Figure 3g). The equivalent vaporization enthalpy of hydrogels is 44% lower than that of pure water, ≈1350 J g^−1^, as shown in Figure 3h. All hydrogels maintain a close vaporization enthalpy due to the similar component ratio of PVA and TA in hydrogels. The evaporation rate and efficiency of different hydrogels (correlative calculation in Section S1.5, Supporting Information) are shown in Figure 3i. Among them, 9—1–C hydrogel shows an optimal water transport, the steadiest network structure, and the highest evaporation rate and efficiency.

### 2.3 Optimized Piezoelectric Fiber Membrane in the Composite Film Evaporator

**Figure 4:**
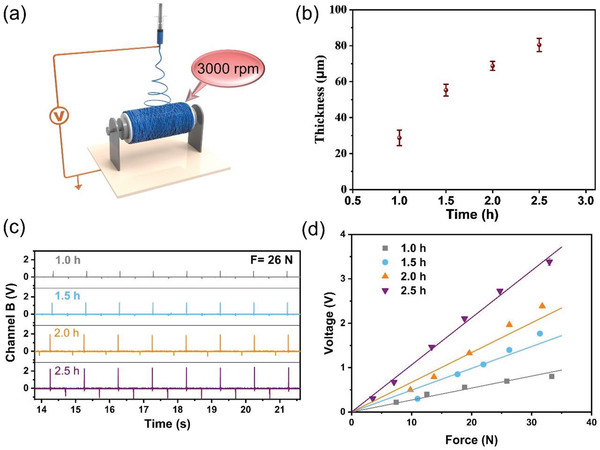
a) Schematic illustration of the preparation of PVDF‐TrFE fibers using the electrospinning method with a high‐speed collecting roller at 3000 rpm. b) Average thicknesses of piezoelectric fiber membranes with different electrospinning time. c) Voltage output of piezoelectric fiber membranes with different electrospinning time under the same sinusoidal mechanical stimulation (26 N). d) Experimental voltage response symbols and corresponding linear fits of piezoelectric fiber membranes with different electrospinning times.

The electrical properties of the piezoelectric fiber membrane play a critical role in the water activation. A high output voltage facilitates the activation process of water, resulting in a higher evaporation rate. The PVDF‐TrFE fibers present a highly anisotropic structure constructed by the traction force of a high‐speed collecting roller at 3 000 rpm during electrostatic spinning (**Figure** **4**a). When the unidirectionality of natural wave spread is taken into account (Figure S4, Supporting Information), for the fiber membrane in the direction perpendicular to wave motion, the ineffective bending will be generated under the wave motion, and thus the wave energy is unable to be converted to the electrical energy. In contrast, the highly anisotropic fibers aligned along the direction of wave motion can be effectively bent in response to wave energy. Consequently, the highly anisotropic fiber membrane is an ideal candidate for effectively responding to wave energy and converting it into electrical energy. Given that the signal output of electrical energy from the piezoelectric fiber membrane is inextricably linked to its thickness, the electrospinning time was controlled to be 1.0 h, 1.5 h, 2.0 h, and 2.5 h to construct piezoelectric fiber membranes with different thicknesses, as shown in Figure 4b.

The electrospinning membrane thickness tends to stabilize when the time exceeds 2.5 h. This phenomenon can be attributed to the weakening of electric fields resulting from the fibrous membrane on the collecting roller, making the subsequent fibers unable to confront the wind generated by the high‐speed rotating collector and spread to the surrounding. To analyze the piezoelectric properties, the as‐prepared fiber membrane was subjected to a sinusoidal mechanical stimulation with a frequency of 1 Hz. The open‐circuit voltage of different electrospinning fiber membranes is shown in Figure 4c. The thickness of the piezoelectric fiber membrane contributes substantially to the electrical properties, and the 2.5 h sample with the thickness of ≈80 µm shows the optimum output signal of 106.1 mV N^−1^. In addition, all the piezoelectric fiber membranes show an excellent linearity (Figure 4d), enabling them to respond to ocean waves containing different energies.

### 2.4 Wave Energy‐Electricity Assessment of the Composite Film Evaporator

**Figure 5:**
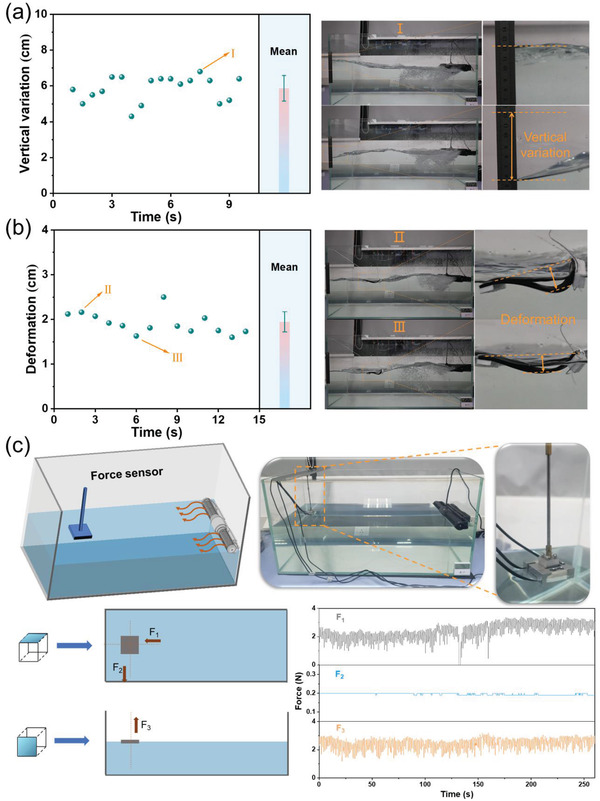
a) Temporal evolution of the vertical variation of waves in the glass tank. Part I shows the photographs of the highest and lowest points of the water surface under a simulated wave. b) Temporal evolution of the deformation of waves in the glass tank. Part II and III show the photographs of the two bending states of the evaporator under a simulated wave. c) Schematic illustration of the prototype for the assessment of impact forces in the water. F_1_, F_2_, and F_3_ represent the forces received by the sensor from three orthogonal directions.

The ocean is a huge natural energy reservoir, in which wave energy is one of the most widely distributed energy sources with the frequency of ordinary gravity ≈0.5–2 Hz.^[^ ^47^ ^]^ Considering the complexity of real ocean waves, an experimental device consisting of wave generation and glass tank were designed to generate waves with the frequency of 1 Hz in this work, which is in favor of evaluating the characteristics of energy conversion and utilization. **Figure** **5**a shows the wave height in the glass tank, and the vertical variation defined as the distance between the highest and lowest positions in the wave motion is ≈5.86 cm. Polystyrene (PS) foams are anchored at both sides of the composite film evaporator to support its self‐floating on the water surface (see details in Section S1.6, Supporting Information). Under the simulated waves, the entire evaporator is bent by the wave impact due to its thin and flexible characteristics (Figure 5b). The deformation defined as the height of bending center protrusion in the evaporator is maintained at a continuous and stable level, namely, ≈1.94 cm. Notably, the evaporator is fixed on the glass tank to enable it to be bent under wave action instead of being directly drifted along with the wave (see details in Section S1.7, Supporting Information).

The impact force in waves is a critical factor to determine the bending behavior of the composite evaporator in the water, and a triaxial force sensor is used to detect and record the impact forces from three orthogonal directions (Figure 5c). To be specific, F_1_ is along the direction of wave generation, and F_2_ and F_3_ follow the directions perpendicular to F_1_ on the water surface and the water surface, respectively. To further assess the impact forces in the water, the positive difference between two adjacent forces was calculated. Notably, some unusual force variations, such as the extremely small fluctuations in the mechanical signal stemming from different wave counteractions and the extremely large signal fluctuations from different wave superimpositions, are excluded from consideration. After calculation, the forces of 1.06 N in F_1_ direction, 0 N in F_2_ direction, and 1.03 N in F_3_ direction are generated. The absence of the mechanical signal in F_2_ direction is due to the unidirectionality of simulated waves. Therefore, the bending behavior of the composite film evaporator in waves with a height of 5.86 cm is obtained, namely, a resultant force of 1.48 N and bending deformation of 1.94 cm.

**Figure 6:**
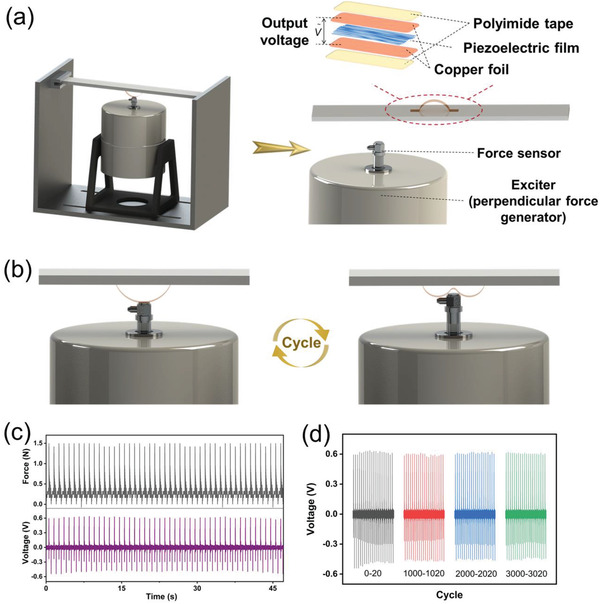
a) Schematic illustration of a prototype used to test the output signal of the piezoelectric fiber membrane. The prototype consists of the exciter, support plate, force sensor, and test sample. The test sample is composed of the piezoelectric fiber membrane, copper foil, and polyimide tape. b) Schematic illustration of the periodic motion of the exciter. c) Voltage output of piezoelectric fiber membrane under the periodic perpendicular displacement of ≈2 cm (purple) and the impact force of 1.5 N (dark grey). d) Voltage output of piezoelectric fiber membrane after different cycles under specific behaviors (a bending deformation of 2 cm and an impact force of 1.5 N).

In order to accurately measure the output signal of the piezoelectric fiber membrane under aforesaid bending behaviors, an experimental device consisting of an exciter, support plate, force sensor, and oscilloscope was designed (**Figure** **6**a). The exciter is applied to deliver a periodic perpendicular displacement of ≈2 cm. The force sensor is employed to monitor the force applied to the sample, which is controlled at ≈1.5 N (see details in Section S1.8, Supporting Information). The sample is fixed on the support plate and attached to the oscilloscope with copper foil. The bending behavior of the sample in the wave is simulated by such a periodic motion device (Figure 6b), and the corresponding piezoelectric output signal is shown in Figure 6c. It is clear that the output signal consists of two characteristic intensities, one originating from the impact of the exciter and the subsequent bending deformation, leading to a strong piezoelectric signal of ≈0.87 V. The other corresponds to the recovery process after the deformation, manifesting a piezoelectric output signal of 0.53 V. Compared to the aforesaid output signals of piezoelectric fiber membrane that was only stimulated by the mechanical impact, the bending of the membrane generates an additional piezoelectric signal output using the designed set‐up, promoting the water activation. In addition, the piezoelectric fiber membrane shows a reproducible and stable output signal after 3000 press‐bend‐release cycles (Figure 6d), demonstrating the remarkable potential for practical long‐term water impact stimulation.

### 2.5 Solar Steam Generation of the Composite Film Evaporator

**Figure 7:**
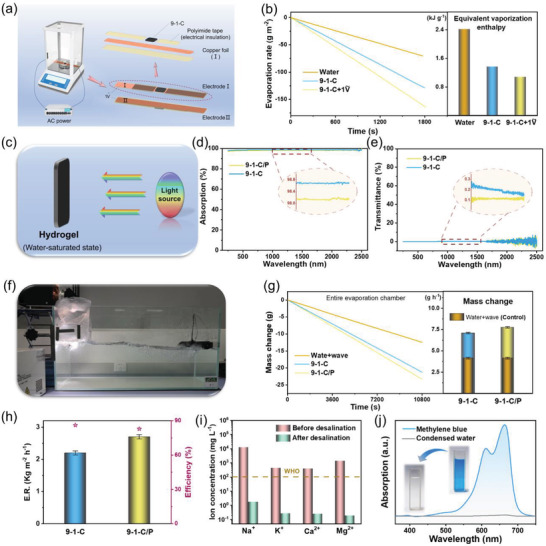
a) Schematic illustration of the prototype used to test actual vaporization enthalpy under an alternating electric field. The prototype consists of the AC power, lab balance, and two equivalent electrodes with an AC voltage of 1 V at 1 Hz. b) Evaporation rate and calculated equivalent evaporation enthalpy of pure water and 9—1–C hydrogels with and without an AC voltage. c) Schematic illustration of the light absorption test. d) Absorptance and e) transparency spectra of the 9—1–C hydrogels with and without piezoelectric fiber membrane. f) Photograph of the prototype used to test the evaporation rate. g) Mass change of entire commercial packaging bag for 9–1–C hydrogels with and without piezoelectric fiber membrane under one sun in comparison to pure water with the waves. h) Average evaporation rate and efficiency of 9—1–C hydrogels with and without piezoelectric fiber membrane. i) Concentrations of four primary ions in standard seawater sample with a salinity of 40 before and after desalination. j) UV‐Vis‐NIR absorption spectra of methyl blue solution and corresponding purified water.

The steam generation performance depends on the energy required during the phase change of water, i.e., equivalent evaporation enthalpy. In our previous work,^[^ ^48^ ^]^ on the basis of different test analysis methods including qualitative analysis, quantitative calculation, and computer simulation, we have demonstrated that the piezoelectric materials can convert the mechanical energy in waves to electrical energy, effectively activating water to reduce the energy required for water evaporation. Similarly, a control experiment using a homemade device was conducted to estimate the actual vaporization enthalpy. Alternating current (AC) power was introduced into the device using two copper foils that were separated by the insulating polyimide tape and placed in the middle of hydrogels (**Figure** **7**a). The device was enclosed in a sealed environment with a stabilized humidity of ≈45% and a temperature of ≈25 °C (see details in Section S1.9, Supporting Information). Accordingly, the equivalent evaporation enthalpy of the water in this system could be estimated by its evaporation rate (see details in Section S1.10, Supporting Information), and a faster evaporation rate means lower equivalent evaporation enthalpy. As shown in Figure 7b, compared to water, the 9—1–C hydrogel exhibits a significant increase in the evaporation rate. Moreover, this evaporation rate was further enhanced when the alternating electric field was applied to the copper foils, demonstrating the ability of the electric field to facilitate water evaporation. Based on these results, the equivalent evaporation enthalpy of water in different states was calculated (Figure 7b). The equivalent evaporation enthalpy of 9—1–C hydrogel is 1.362 kJ g^−1^, which decreases to 1.077 kJ g^−1^ when applying an alternating electric field intensity of 1 V. That is, the energy required for its evaporation is significantly less than that for natural water evaporation with an evaporation enthalpy of 2.413 kJ g^−1^, verifying the efficient water activation capacity of the electric field.

Carbon‐based materials are excellent light absorbers that possess excellent photo absorption over the whole solar wavelengths.^[^ ^49^, ^50^, ^51^, ^52^, ^53^ ^]^ Graphene oxide (GO) in the hydrogel was reduced by TA at 10 °C to obtain reduced GO (rGO), which served as the light absorber to allow the entire evaporation system to perform the photothermal conversion. The ultraviolet‐visible near‐infrared (UV‐vis‐NIR) spectra were used to investigate the absorptivity and transmittance of 9—1–C hydrogel and 9—1–C hydrogel with piezoelectric fiber membrane (9—1–C/P). To accurately evaluate the light absorption characteristics of hydrogels during the practical evaporation process, the hydrogels were tested in a water‐saturated state (Figure 7c). As shown in Figure 7d,e, owing to the opaqueness and reflectivity of the piezoelectric fiber membrane, 9–1–C/P hydrogel exhibits a slight decrease in light absorption and transmittance compared to the non‐piezoelectric 9–1–C hydrogel. Nonetheless, the integrated hydrogel shows a broad absorptance of ≈98% in the sunlight wavelength region of 250–2500 nm.

The steam generation performance of the composite film evaporator was examined by the evaporation rate of water under one sun. To obtain an accurate evaporation rate of the composite evaporator under simulated waves, the entire system was placed in a commercial packaging bag that can move with the waves (Figure 7f). Similar to the aforementioned wave height analysis in the glass tank, a wave energy retention of ≈96.4% with a vertical variation of 5.65 cm was observed (Figure S5, Supporting Information), indicating that there is no significant effects on energy intensity contained in the artificial wave after using the bag in the evaporation system. The mass change of water inside the bag was used to evaluate the evaporation rate, and the mass change in the absence of the evaporator was subtracted as a control (Figure 7g). As shown in Figure 7h, the calculated evaporation rates of water for 9—1–C and 9—1–C/P hydrogels were ca. 2.197 ± 0.063 and 2.702 ± 0.066 kg m^−2^ h^−1^, respectively. The evaporation rate of 9—1–C sample remained consistent with the foregoing hydrogel structure optimization analysis (Figure 3i), indicating the reliability of the evaporation rates obtained using a commercial packaging bag. In addition, 9—1–C/P hydrogel shows a 23% increase in evaporation rate compared to 9–1–C hydrogel owing to that the piezoelectric fiber membrane efficiently converts wave energy to electrical energy, activating water in adjacent regions and accelerating water evaporation. Combining the evaporation rate with the aforementioned equivalent evaporation enthalpy, both hydrogels with and without piezoelectric fiber membrane exhibit evaporation efficiencies greater than 80% (Figure 7h).

Furthermore, a spherical chamber was designed to condense and collect the water vapor, followed by evaluating the water purification ability. The spherical chamber contains a hemispherical shell with standard brine (salinity of 40), which can move with the waves (Figure S6, Supporting Information). Under one‐sun illumination, the brine evaporated and concentrated on the walls, eventually converging at the bottom of the spherical chamber. Inductively coupled plasma optical emission spectroscopy (ICP‐OES) was applied to evaluate the quality of the collected water, as shown in Figure 7i. The concentrations of the primary ions (Na^+^, K^+^, Ca^2+^, and Mg^2+^) in standard brine and concentrated water were detected, all of which were reduced by 3–4 orders of magnitude. The ionic concentrations of purified water were significantly lower than the drinking water guidelines set by the World Health Organization (WHO).^[^ ^54^, ^55^ ^]^ In addition, methyl blue dye as simulated wastewater was used to analyze the decontamination performance of the composite film evaporator using UV‐vis‐NIR spectra. As shown in Figure 7j, the blue liquid turns completely clear after the water purification process, giving rise to an ≈100% decontamination efficiency.

## 3 Conclusion

A brand‐new solar‐steam generator comprising piezoelectric film and hydrogel is capable of synergistically activating water for accelerating evaporation rate, in which a piezoelectric fiber membrane attains an efficient wave‐driven electrical energy conversion. We precisely evaluated the wave energy and the motion behavior of the evaporator in the wave, and subsequently tested and predicted the signal output of the flexible piezoelectric fiber membrane under the wave using a homemade experimental device. The piezoelectric fiber membrane generates a high voltage output of 0.87 V, enabling electro‐driven reactivation to promote water evaporation. In a solar steam generation, the composite film evaporator featuring the dual water activation functionality originating from electrical energy and hydrogel delivers a high steam generation rate of 2.702 kg m^−2^ h^−1^ under one‐sun illumination (1 kW m^−2^), where electrical energy activation leads to an improvement of over 23% compared to a non‐piezoelectric hydrogel evaporator. This strategy offers a promising evaporation prototype based on the synergistic activation of electrical energy and hydrogel. More significantly, the superiorities in long‐term durability, desalination, and purification performance, as well as the tolerance of complex contaminants are beneficial for promoting the industrialization of solar vapor generation.

## 4 Experimental Section

### Materials

PVA with a weight‐average molecular weight of 146 000—186 000 and TA with a number‐average molecular weight of 1701.2 were purchased from Aldrich and Adamas‐beta, respectively. N, N‐dimethylformamide (DMF, ≥ 99.5%) and acetone (≥ 99.5%) were supplied by Xilong Science Co., Ltd. (Chengdu, China). GO with a diameter of 10–50 µm and a thickness of 0.5–3 nm was purchased from Technology Co., Ltd. (Suzhou, China). Glycerol (≥99.0%) was supplied by Fuchen Chemical Reagent Co., Ltd. (Tianjin, China). Piezoelectric copolymer PVDF‐TrFE powders (75:25 mol%) were purchased from Arkema. All the chemicals were used without further purification.

### Fabrication of the Piezoelectric Fiber Membrane

First, 0.75 g PVDF‐TrFE powders were dissolved in 5 mL DMF/acetone binary solution with a volume ratio of 3:2 at 25 °C with magnetic stirring at 300 rpm until the powers were completely dissolved. The solution was sucked into a 5 mL syringe with a stainless steel needle with an inner diameter of 0.41 mm and then ejected at a flow rate of 0.6 ml h^−1^ and an accelerating voltage of 12 kV. The collecting roller covered by aluminum foil was controlled at a high rotating speed of 3000 rpm to collect the PVDF‐TrFE fibers. Finally, the resulting fiber membrane was placed in an oven at 60 °C for 24 h to remove residual solvent.

### Fabrication of PVA/TA Hydrogels with Different Ratios of Water to Glycerol

First, 100 mg GO powders were added in 50 mL deionized (DI) water/glycerol binary solution with the ultrasonic treatment for 30 min to obtain a uniform suspension. The ratio of DI water to glycerol varied from 5:5 and 7:3 to 9:1. Subsequently, 5 g PVA and 1.25 g TA were dissolved in the aforementioned suspension at 100 °C with magnetic stirring at a speed of 200 rpm, followed by pouring the solution into a glass mold for the gelation at −20 °C for 12 h. The obtained hydrogels were abbreviated as 5–5, 7—3, and 9–1 according to the ratio of DI water to glycerol and immersed into DI water to fully swell for subsequent measurements. In addition, the solution with the DI water/glycerol ratio of 9:1 was poured into polytetrafluoroethylene (PTFE) mold placed on a metal support frame with the bottom immersed in liquid nitrogen (Figure S7, Supporting Information). The solution was frozen at ‐100 °C with ambient temperature of −30 °C for 2 h and then stored at −20 °C for 12 h to prepare the hydrogel with aligned channel structure. The obtained hydrogel denoted as 9—1–C was immersed into DI water to fully swell for subsequent measurements.

### Fabrication of the Composite Film Evaporator

Based on optimization results, the ratios of TA to PVA and water to glycerol were controlled at 1:4 and 9:1 to fabricate the piezoelectric composite film evaporator. The as‐prepared piezoelectric fiber membrane was cut into a rectangle with a dimension of 3.5 × 8.5 cm^2^. The fiber membrane and PVA/TA solution containing GO were transferred into a PTFE mold, followed by freeze‐casting and swelling described above to obtain the composite film evaporator with a thickness of 2 mm.

### Equivalent Water Vaporization Enthalpy of Hydrogels

The evaporation behavior of hydrogels was investigated using a DSC Q20 (TA Instruments, Milford, MA, USA) under a nitrogen atmosphere. The hydrogel sample was placed in a closely sealed Al crucible and measured at a heating rate of 5 °C min^−1^ from 40 to 200 °C under a nitrogen flow flux of 50 ml min^−1^.

### Test System for the Steam Generation

The experimental system for water evaporation performance consists of a solar simulated device (CEAULIGHT, CEL‐HXUV300 xenon lamp, China) with an optical filter (standard AM 1.5 solar spectrum), an optical detector (PerfectLight, PL‐MW2000 optical power meter, China), water pump (55 W), glass tank with a size of 80 × 35 × 40 cm^3^, commercial packaging bag with a size of 31 × 51 cm^2^, and lab electronic balance with an accuracy of ± 0.1 mg (OHAUS PX523ZH/E, China). The solar flux was measured and controlled at one sun (1 kW m^−2^) using an optical detector. The evaporator was placed in a commercial packaging bag, and the mass change of the entire commercial packaging bag was measured using a balance to obtain the evaporation rate.

### Test System for the Electricity Output

The piezoelectric properties of piezoelectric fiber membranes were characterized under sinusoidal mechanical stimulation generated using an exciter (TJZ 20, Test Co. Ltd., China). The applied force was detected and controlled using a quartz force sensor (208C02, PCB Piezotronics, USA). Piezoelectric output signals were recorded using an oscilloscope (Pico Technology, USA). The alternating electric field in the actual vaporization enthalpy estimation was provided by a bench signal generator (DG1032Z, Rigol Technology Co., Ltd, China).

### Characterization of the Composite Film Evaporator

The morphologies of the membranes were characterized using a scanning electron microscope (SEM, Nova Nano 450, USA) equipped with an EDS detector. Tensile analysis was measured at room temperature on a universal testing machine (Instron 5967, USA) at a loading rate of 100 mm min^−1^. The dynamic mechanical analyses were measured using an advanced rheometer (Discovery HR20, TA Instruments, USA). The impact forces in the water were detected using a triaxial force sensor (DY500, Dayang Sensor System Engineering Co., Ltd., China). The absorption and transmittance spectra of the composite film evaporator were recorded on a UV‐vis‐NIR spectrophotometer (Shimadzu UV3600, Japan) equipped with an integration sphere unit. The ion concentrations of the brine and collected water were tracked using an inductively coupled plasma optical emission spectrometer (ICP‐OES, Agilent 5100 SVDV, USA).

## Conflict of Interest

The authors declare no conflict of interest.

## Supporting information

Supporting Information

## Data Availability

The data that support the findings of this study are available from the corresponding author upon reasonable request.
