# Supplementary material for: A Wave‐Driven Piezoelectrical Film for Interfacial Steam Generation: Beyond the Limitation of Hydrogel
Source: Adv Sci (Weinh). 2022 Oct 10;9(33):2204187. doi: 10.1002/advs.202204187 (PMC9685475; doi:10.1002/advs.202204187)
Supplement: Supplementary file 1 — Supporting Information [file ADVS-9-2204187-s001.pdf]

ADVANCED SCIENCE
Open Access

Supporting Information

for *Adv. Sci.*, DOI 10.1002/advs.202204187

A Wave-Driven Piezoelectrical Film for Interfacial Steam Generation: Beyond the Limitation of Hydrogel

Sen Meng, Chun-Yan Tang, Jie Yang, Ming-Bo Yang and Wei Yang**

Electronic Supplementary Information (ESI)

A wave-driven piezoelectrical film for interfacial steam generation: Beyond the limitation of hydrogel

Sen Meng, Chun-Yan Tang, Jie Yang*, Ming-Bo Yang, Wei Yang*

College of Polymer Science and Engineering, Sichuan University

State Key Laboratory of Polymer Materials Engineering

Chengdu, 610065, Sichuan, China.

E-mail: psejieyang@scu.edu.cn (J. Yang); weiyang@scu.edu.cn (W. Yang)

Section S1.1 Optimization of tannic acid in the composite film evaporator

Tannic acid (TA) can increase the mechanical property and stability of the polyvinyl alcohol (PVA) network through massive hydrogen bonds.[1] To analyze the effect of TA on the properties of hydrogels, PVA hydrogels with different TA contents (TA-to-PVA mass ratios of 1:4, 1:2, and 1:1) were prepared and abbreviated as 1-4, 1-2, and 1-1, respectively. In addition, the PVA hydrogel without TA prepared by the same method was abbreviated as 0. The saturated water content in PVA hydrogels decreases sharply with increasing TA content (Figure S8a), and the low water content cannot meet the water supply during evaporation, which significantly limits its evaporation rate. As shown in Figure S8b and c, the equivalent water vaporization enthalpy of hydrogels was measured through DSC measurement. The substantial hydrophilic functional groups from TA induce strong interactions with water molecules, reducing the overall energy demand of vapor generation.[2] Based on these considerations, the 1-4 hydrogel featuring a high water content exhibits a low vaporization enthalpy.

Section S1.2 Pore structure analysis of hydrogels

The molds used in the gelation process of hydrogels are made of glass and polytetrafluoroethylene (PTFE), which generally present a highly hydrophobic surface. On the contrary, PVA exhibits highly hydrophilic behaviors due to its substantial hydroxyl functional groups, and this mismatched surface properties are detrimental to the stretchability of PVA molecular chains on the mold surface, resulting in a reduced pore size on hydrogel surfaces. To increase the pore size on the hydrogel surface, a

commercial anti-fogging agent was used to modify the mold surface characteristic. Contact angle measurements were conducted to investigate the water contact behavior of the mold surface using a video optical contact angle analyzer (KRüSS DSA25, GER) with an accuracy of 0.1 °. Figure S9 shows the water contact angle results for glass and PTFE molds with and without commercial anti-fogging agent, and it can be clearly seen that the mold surface exhibits super-hydrophilic behavior with a contact angle of 0 ° after the hydrophilic modification. Moreover, the 9-1 hydrogel prepared using the treated mold maintains a uniform pore size throughout the sample (Figure S3), facilitating water transportation process.

Section S1.3 Investigation of the water transport performance of PVA hydrogels

The detailed water transporting performance of hydrogels with different water/glycerol ratios is shown in Figure S10. Commercial cotton cores with a diameter of 7 mm and a length of 13 cm are used as standard water absorbent, and the methyl blue dye is used for visual contrast (Figure S10a). After the cotton core is placed into the dye, water can be quickly absorbed into it by capillary action, enabling a height of 6 cm (Figure S10b). Subsequently, this transport process tends to be stabilized and the height of dye remains constant after absorption for 3 h (Figure S10c). For the hydrogels with different water/glycerol ratios, the water absorption capacity showed significant variability, as shown in Figure S10d. The 5-5 hydrogel with smallest pore size is unable to absorb water into the cotton core due to its poor water transfer capability. The 9-1 hydrogel with large pore size exhibits an enhanced water transfer capacity, but they still cannot meet the

water supply demand for high-speed water evaporation. A large pore structure oriented along the out-of-plane direction of the evaporator significantly increases the water transfer capacity for 9-1-C hydrogel treated with additional ice-templating control process, giving rise to the high-speed and efficient solar interfacial vapor generation.

Section S1.4 Investigation of the crystallization behavior of PVA hydrogels

The crystallization behavior of hydrogels was investigated using a DSC under a nitrogen atmosphere. The hydrogel sample was placed in a closely sealed Al crucible and measured from 30 to 260 °C with a heating rate of 5 °C min^{-1} under a nitrogen flow flux of 50 ml min^{-1}. The typical DSC melting curves of hydrogels with different water/glycerol ratios and 9-1 hydrogel prepared by the additional ice-templating operation were shown in Figure S11. The enthalpy change of aforementioned hydrogels were calculated, as listed in Table S1. Higher enthalpy change indicates that more crystalline domains are formed, verifying the promoting effect of the extremely low temperature on PVA crystallization.[3-5]

Section S1.5 Evaporation efficiency calculation of PVA hydrogels

The evaporation efficiency was calculated from the vapor generation rate (η), which is expressed by equation (1):

$$\boldsymbol{\eta} = \frac{\boldsymbol{v h_w}}{\boldsymbol{C_{opt} p_n}} \quad (1)$$

Where υ is the vapor generation rate, h_w is the equivalent evaporation enthalpy of hydrogels obtained by DSC measurements (Figure 3h), C_{opt} is the optical concentration, and p_n is the normal illumination intensity of one sun (1 kW m^{-2}). The calculated

evaporation efficiencies of hydrogels for 5-5, 7-3, 9-1, and 9-1-C were 36.28%, 49.13%, 63.42%, and 89.33%, respectively.

Section S1.6 Assembly of floating layer in the composite film evaporator

Polystyrene (PS) foams were anchored at both sides of the composite film evaporator to support its self-floating on the water surface (Figure S12). Each PS foam was fixed to the evaporator by two thumbtacks that were inserted from the bottom to the top of the evaporator. Considering the direction of wave impact in water (Figure 5c), the impact force generated by the waves carries a component perpendicular to the water surface. Thus, this fixed method will be self-tensioned without detaching owing to the action of waves.

Section S1.7 Deformation measurement of composite film evaporator in waves

In order to ensure the bending effect of the evaporator under the wave impact, the evaporator is fixed on the glass tank to avoid being directly drifted along with the wave. Two thermoplastic polyurethane (TPU) wires tightly sandwiched between the PS foam and the evaporator were fixed to the rim of glass tank, which significantly limited the movement of the evaporator along with the waves (Figure S13). In addition, the irradiated area of the simulated sunlight is limited for steam generation performance tests of the composite film evaporator. Therefore, the evaporator was also fixed on the glass tank to ensure the illumination and conversion of the incident sunlight.

Section S1.8 Force control in piezoelectric performance measurement of fiber membranes

In the wave generated impact test, the calculated resultant force acting on the evaporator is 1.48 N (Figure 5c). Herein, the force acting on the piezoelectric fiber membrane needs to be controlled to ensure the accuracy of the piezoelectric performance test. Nevertheless, no force signal is observed for the fiber membrane when bending due to the low air resistance. A plastic spacer is placed on the backside of the impact surface of the fiber membrane to provide bending resistance, and the force sensor is applied to ensure that the fiber membrane is subjected to a force of 1.5 N.

Section S1.9 Environmental control of the device for testing the actual vaporization enthalpy

The entire device was enclosed in a sealed lab balance, in which a supersaturated potassium carbonate solution was used to maintain a stable relative humidity of *ca.* 45%. The room temperature was maintained at *ca.* 25 °C. The balance was covered with an opaque black-out cloth to eliminate effects of ambient light on the result.

Section S1.10 Estimation of the equivalent vaporization enthalpy

To evaluate the energy required for hydrogel under an alternating electric field, a control experiment was designed to estimate the actual vaporization enthalpy. The device was enclosed in a sealed environment with a stabilized humidity of *ca.* 45% and a temperature of *ca.* 25 °C. Accordingly, the equivalent evaporation enthalpy (E_{equ}) of the hydrogel in this system could be estimated by its evaporation rate, which is expressed by equation (2):

$$U_{in} = E_1 m_1 = E_{equ} m_2 - U_{ele} \quad (2)$$

where U_{in} is the total external energy input, E_1 is the evaporation enthalpy of pure water obtained by DSC measurements (Figure 3g, 2413 J g^{-1}), m_1 and m_2 are the mass changes of pure water and the hydrogel with an AC voltage, and U_{ele} is the external electrical energy input.

Given that the electrical energy is derived from the piezoelectric fiber membrane in place of external energy in practical applications, U_{ele} can be regarded as 0 to simulate the water evaporation in such a piezoelectric material-based composite film evaporator. In addition, the hydrogel without an AC voltage was also evaluated for the equivalent evaporation enthalpy using the same method.

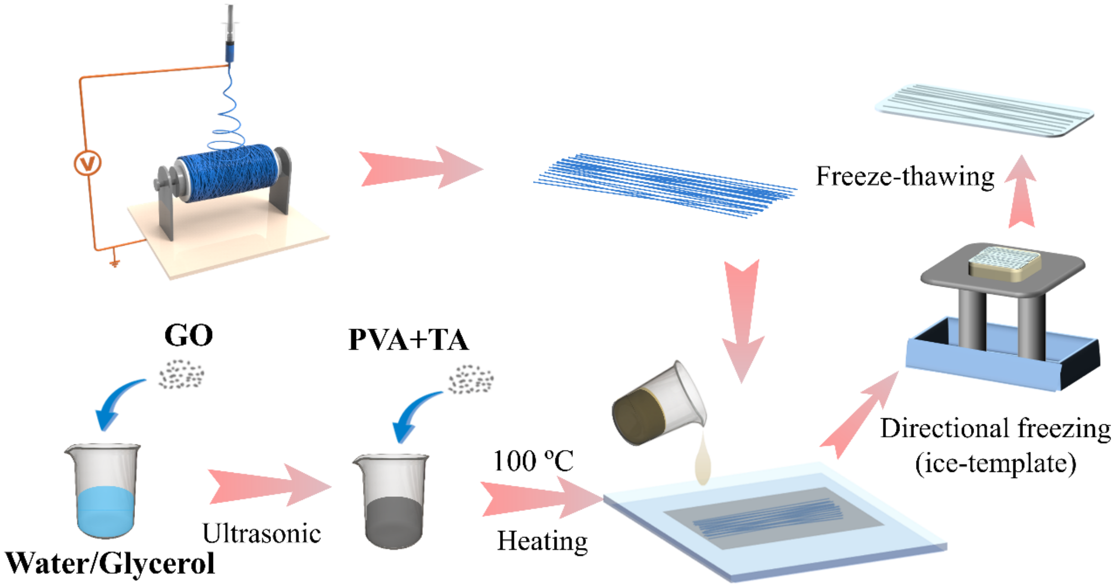

Figure S1 Schematic of the preparation process of the composite film evaporator.

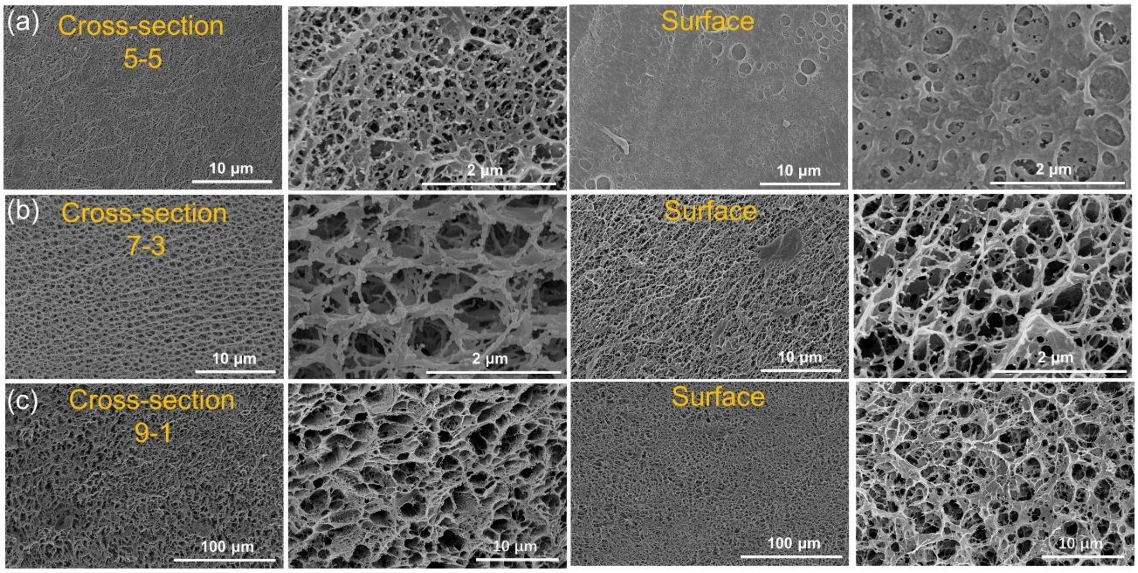

Figure S2 Cross-sectional and surface SEM images of (a) 5-5, (b) 7-3 and (c) 9-1 hydrogels.

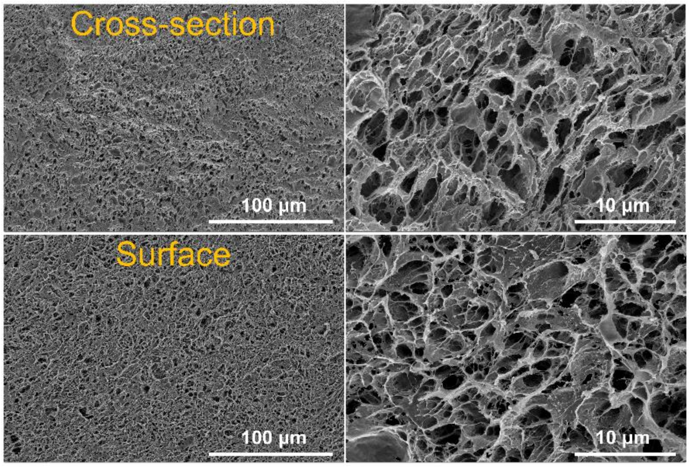

Figure S3 Cross-sectional and surface SEM images of the 9-1 hydrogel prepared using the glass mold with commercial anti-fogging agent.

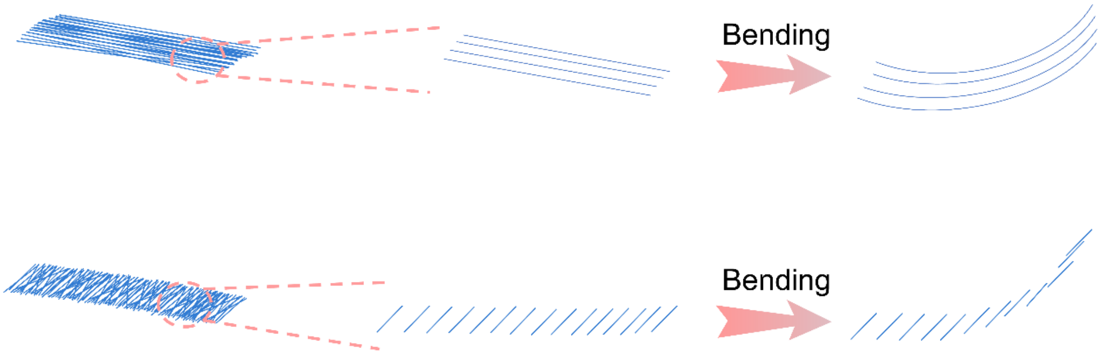

Figure S4 Schematic of piezoelectric fiber membranes with different anisotropic arrangements during bending process.

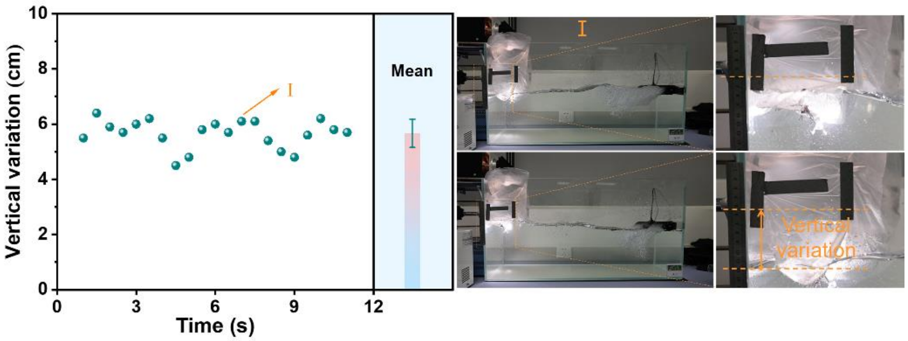

Figure S5 Temporal evolution of the vertical variation of waves in a commercial packaging bag placed in the glass tank. I shows the photograph of the highest and lowest points of the water surface in the commercial packaging bag under the simulated wave.

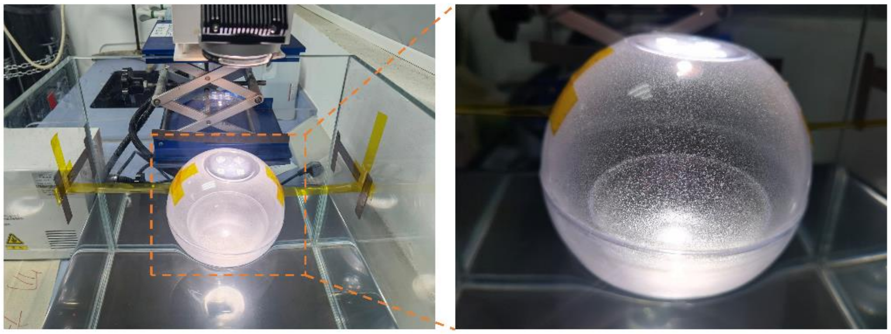

Figure S6 Photographs of a floating water-harvesting prototype.

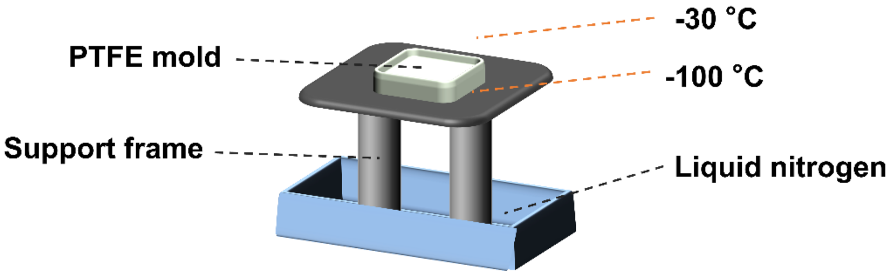

Figure S7 Schematic of the ice-templating-assisted directional freezing strategy.

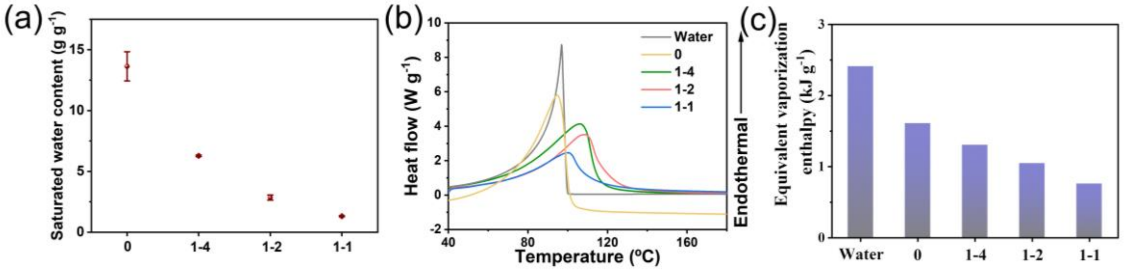

Figure S8 (a) Saturated water content of hydrogels with different TA/PVA ratios. (b) Typical DSC melting curves (c) Equivalent vaporization enthalpy of pure water and hydrogels with different TA/PVA ratios.

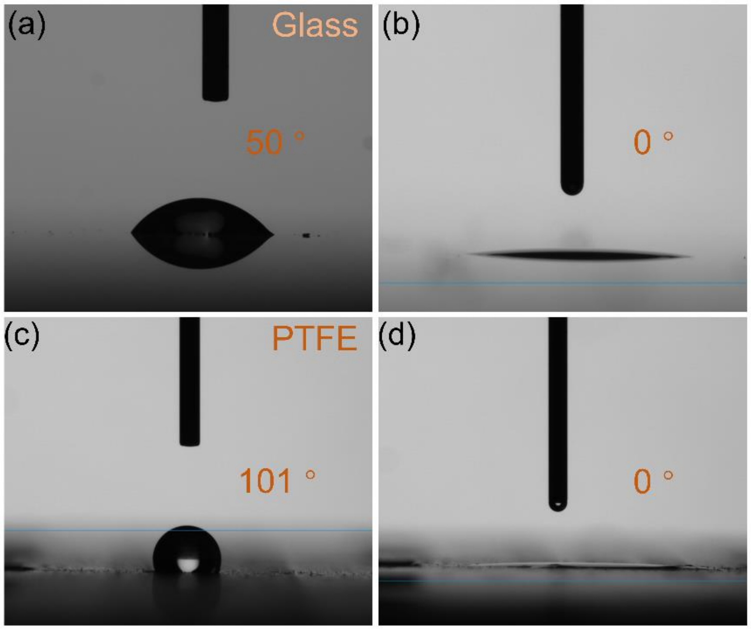

Figure S9 Contact angle measurement diagrams for glass mold (a) with and (b) without commercial anti-fogging agent. Contact angle measurement diagrams for PTFE mold (c) with and (d) without commercial anti-fogging agent.

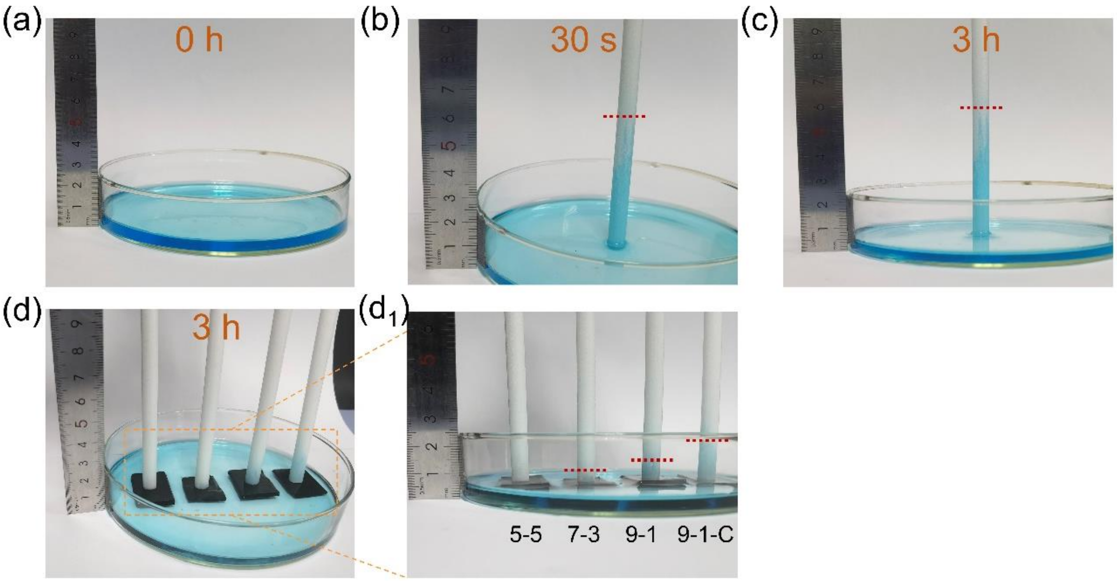

Figure S10 (a) Photographs showing the setup of the water transport using methyl blue dye with a concentration of 20 mg L^{-1}. Time-dependent water transporting images of commercial cotton cores (b, c) without and (d) with hydrogels.

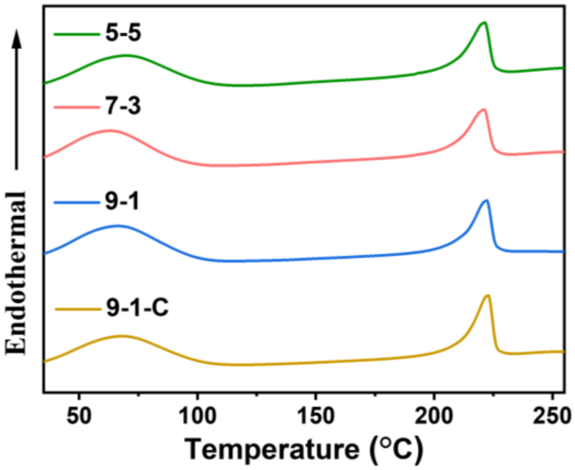

Figure S11 Typical DSC melting curves of hydrogels with different water/glycerol ratios and 9-1-C hydrogel fabricated by additional ice-templating operation.

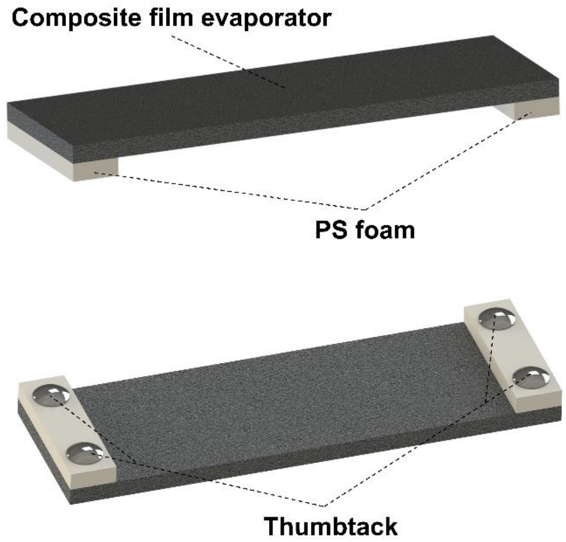

Figure S12 Schematics of the assembly of floating layer in composite film evaporator.

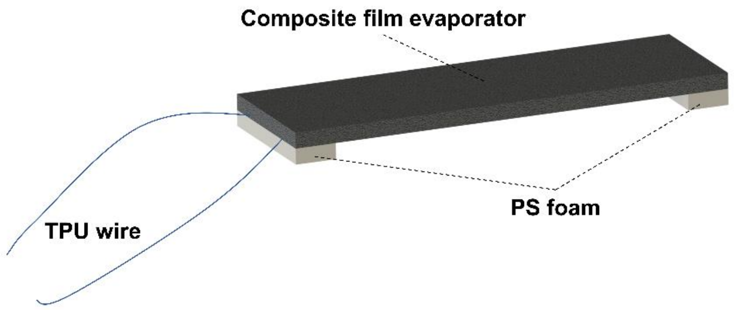

Figure S13 Schematic of TPU wire assembly in composite film evaporator.

Table S1 Enthalpy change of different samples

Hydrogels	*Enthalpy change (J g^{-1})*
**5-5**	**64.07**
**7-3**	**58.38**
**9-1**	**56.11**
**9-1-C**	**61.23**

References

[1] W. Chen, N. Li, Y. Ma, M. L. Minus, K. Benson, X. Lu, X. Wang, X. Ling, H. Zhu, *Biomacromolecules* **2019**, 20, 4476.

[2] X. Zhou, F. Zhao, Y. Guo, B. Rosenberger, G. Yu, *Sci. Adv.* **2019**, 5, eaaw5484.

[3] C. M. Hassan, N. A. Peppas, *Macromolecules* **2000**, 33, 2472.

[4] X. J. Zha, S. T. Zhang, J. H. Pu, X. Zhao, K. Ke, R. Y. Bao, L. Bai, Z. Y. Liu, M. B. Yang, W. Yang, *ACS Appl. Mater. Interfaces* **2020**, 12, 23514.

[5] H. Adelnia, R. Ensandoost, S. Shebbrin Moonshi, J. N. Gavgani, E. I. Vasafi, H. T. Ta, *Eur. Polym. J.* **2022**, 164, 110974.
